# COVID-19 in a shattered health system: Case of Libya

**DOI:** 10.7189/jogh.11.03058

**Published:** 2021-04-03

**Authors:** Godsgift Chinemelum Iwendi, Alkamel Mohamed Alsadig, Mashkur Abdulhamid Isa, Amos Abimbola Oladunni, Mohamed Babiker Musa, Attaullah Ahmadi, Yusuff Adebayo Adebisi, Don Eliseo Lucero-Prisno

**Affiliations:** 1 Faculty of Pharmaceutical Sciences, University of Port Harcourt, Rivers State, Nigeria; 2 Tripoli Military Hospital, Libya; 3 School of Health and Related Research, University of Sheffield, United Kingdom; 4 Faculty of Pharmaceutical Sciences, Ahmadu Bello University, Zaria, Nigeria; 5 Faculty of Pharmacy, Omdurman Islamic University, Khartoum, Sudan; 6 Medical Research Center, Kateb University, Kabul, Afghanistan; 7 Faculty of Pharmacy, University of Ibadan, Ibadan, Nigeria; 8 Department of Global Health and Development, London School of Hygiene and Tropical Medicine, London, UK; 9 Faculty of Management and Development Studies, University of the Philippines (Open University), Los Baños, Laguna, Philippines.

The first case of COVID-19 was identified in Wuhan city, China in December 2019. Since then, it has been ravaging many countries across the globe [1], including African countries [2]. Libya is one of the countries that was affected by the crisis. As of 21st December 2020, there were a cumulative total of 95 708 cases and a total of 1385 deaths [3].

The first case of COVID-19 in Libya was detected on 14th March 2020 in an elderly man who was visiting the country from Saudi Arabia [4]. The government had declared COVID-19 as a public health emergency on 14th March 2020 in an attempt to stop the importation of the virus [5]. On March 17, several social and public health measures were put in place including the closure of the country’s borders, flight suspension, travel ban for foreign nationals, closure of schools, universities, cafes, restaurants and mosques. On March 30, the government announced the release of 466 detainees in Tripoli in a bid to halt the spread of the virus among prison inmates [6].

While the government has responded well to the pandemic, the escalating armed conflict has made it difficult to contain the spread of the virus [7]. The ongoing border fight has led to unnecessary loss of lives, displacement and destruction of vital infrastructure thereby resulting in unsafe living conditions with limited access to health care, essential medicines, food, shelter, education and unsafe drinking water [7]. More so, local and international humanitarian organizations have responded by providing emergency relief operations to internally displaced people (IDP), returnees, conflict-affected non-displaced Libyans including migrants and refugees who are affected by COVID-19 or on-going insecurity [8], but there were challenges of access as a result of depletion of humanitarian spaces available for relief societies [9].

According to the International Displacement Monitoring Center, a total number of 45 100 people were living in internal displacement as a result of conflict and violence by 31st December 2019 [6]. This situation makes it difficult to conduct an actual situational analysis of COVID-19 impact in the population. To this end, we describe the current situation of the pandemic in Libya and argue the need for immediate public health response to COVID-19.

## POLITICAL INSTABILITY

Libya has been in an unceasing state of political unrest in the last decade, starting with a series of protests and uprising which led to the end of the Qaddafi regime in 2011 [10]. The country was thrown into a state of destabilization following his demise, as conflict erupted between different tribal, militant and political groups, each seeking to seize power and this led to a civil war in 2014 [10]. An election in August 2014 saw the House of Representatives (HoR) emerge victoriously but this was met by opposition from the General National Congress (GNC) [8]. More violence ensued with General Khalifa Haftar of the Libyan National Army (LNA) appointed as the commander by forces loyal to the HoR, thereby taking control of the East [8].

The international community including the UN have intensified efforts over the years to see the formation of a unified government through negotiations, peace talks and formation of an agreement involving different parties envisaging the Government of National Accord (GNA). These efforts have mostly failed with the LNA continually seeking political and military control, thus, making unrelenting violence the norm in Libya [8]. Political instability in Libya has thrust the country into a state of insecurity with an ever-worsening humanitarian crisis [8,10,11]. Like war-affected countries experiencing disruption of public health interventions [12], the socio-economic structure and health care system of Libya is already severely weakened by the war which together put the country at an incredibly vulnerable situation during the COVID-19 pandemic [10,13].

## INCREASED VULNERABILITY TO SARS-COV-2 TRANSMISSION

Libya has been ranked among the high-risk countries for COVID-19 in the region by the World Health Organization (WHO). Access to health care in has been impacted in many areas by the ongoing war, resulting in limited laboratory testing capacity for COVID-19 (with only two laboratories in Tripoli and Benghazi) [5]. Limited monitoring of contracts, logistical challenges in accessing a large part of the population, and stigmatization have resulted in people not accessing treatment when ill. Many health centres have closed in the aftermath of the announcement of first cases and lockdown notification. There was a shortage of medications, equipment, and personnel to provide essential services in available facilities [7]. Civilian infrastructure, resulting in water and electricity cuts and leaving people without access to facilities, has also been intentionally targeted by warring sides [10]. Hospitals continue to be struck by shelling, destroying the completely operational 400-bed hospital, the 400-bed Al-Khadra General hospital in Tripoli was destroyed in early April [10].

**Figure:**
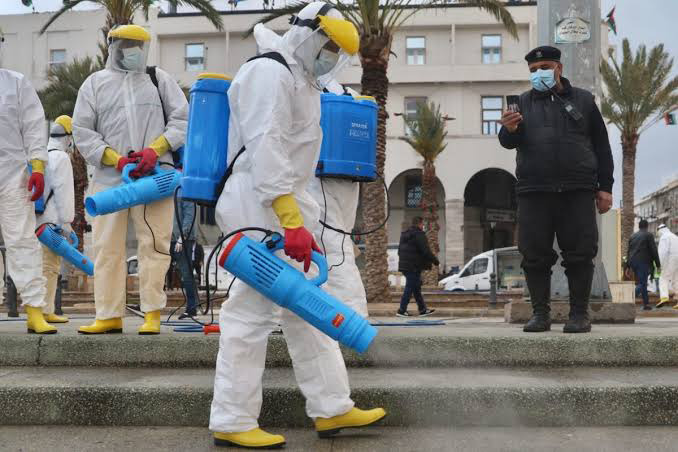
Photo: Officials carry out disinfection works within coronavirus (COVID-19) precautions at Matyrs’ Square in Tripoli, Libya on 25 March, 2020 (Hazem Turkia / Anadolu Agency). From https://www.middleeastmonitor.com/20200610-coronavirus-cases-surge-in-libya-after-repatriations/amp/ (copyright free).

Over 24 000 people have been displaced as a result of the war. Most of them live in overcrowded conditions that allow the virus to spread rapidly. The health system in Libya is near collapse, with three-quarters of primary health care facilities not working due to shortages of medical workers, supplies, medication, and equipment [11]. Shortages have made it difficult to determine the effect on the population of COVID-19. Surveillance is a key component of public health practice as it involves the monitoring of the spread of a disease. However, the lack in number of “sentinel sites” (health facilities responsible for monitoring and detecting the occurrence of specific diseases), in Libya, means that infections can go undetected, allowing COVID-19 to propagate within the community thereby making the spread of COVID-19 difficult to control [7,11].

## FOOD AND FINANCIAL INSECURITIES DURING THE COVID-19 PANDEMIC

During the COVID-19 pandemic, food security has been a global concern [14,15], Libya has been facing acute food and financial insecurities with more than 7% of the Libyan population at risk with the latest conflict raging between April 2019 and May 2020 during the COVID-19 pandemic [10]. Food staples (wheat, bread, flour, oil and rice) have been available in markets especially staple food commodities such as wheat bread, flour, oil, and rice but prices have been highly volatile, more so for imported food items, which include most processed commodities [7]. The lack of cash during the pandemic and delayed salaries on top of long-standing financial problems has also contributed to increased prices and lower purchasing power of citizens during the pandemic [7].

## IMMEDIATE PUBLIC HEALTH RESPONSE REQUIRED

With the WHO classifying Libya amongst high-risk countries in Africa [7], some immediate public health responses are required, this includes increasing the capacities for testing and isolation centres through social mobilization and strong community engagement via adequate sensitization and awareness strategies to overcome stigmatization.

Additional health professionals need to be engaged in community health centres that are providing care to the rural and politically unstable areas. They should be adequately equipped with personal protective equipment (PPE) and hand sanitisers etc. with support from donations by the international communities as well as humanitarian agencies [9].

The evacuation and resettlement of refugees, asylum and migrants from various detention centres would also play a major role in decreasing the spread of the coronavirus due to the over-crowded conditions in these centres [10]. Activities of the Libya Governance and Civil Society Program (LGCS) and Libya Elections and Legislative Strengthening Activity (LELSA), Libya Public Financial Management (LPFM) should be encouraged to maintain peace, economic stability and good governance in the country [9].

## CONCLUSION

The combination of the pandemic alongside crisis, political instability and violence in Libya further emphasizes the vulnerability of the country’s populace. The COVID-19 measures such as lockdown and restrictions in the movement leading to poor access to health centers and health professionals have also contributed negatively to the already shattering health system of the country. Without targeted efforts and initiatives such as community engagements, good governance and international bodies that address all aspects of these crises, COVID-19 will continue to cause more harm to the country and the world at large.
